# Multiorgan Oxidative Damage and Gene Expression Modulation in *Danio rerio* Induced by Pollutants from the Tepetitlán Reservoir, Mexico

**DOI:** 10.3390/ijms27010075

**Published:** 2025-12-21

**Authors:** Nely San Juan-Reyes, Leobardo Manuel Gómez-Oliván, José Manuel Orozco-Hernández, Eduardo Javier Quiroz-Fabela, Francisco Escobar-Huerfano, Karina Elisa Rosales-Pérez, Gustavo Axel Elizalde-Velázquez, Sindy San Juan-Reyes, Selene Elizabeth Herrera-Vázquez

**Affiliations:** 1 Laboratorio de Toxicología Ambiental, Facultad de Química, Universidad Autónoma del Estado de México, Paseo Colón intersección Paseo Tollocan, Colonia Residencial Colón, Toluca CP 50120, Mexico; 2 Departamento de Neonatología, Hospital de Ginecología y Obstetricia No. 221, Instituto Mexicano del Seguro Social (IMSS), Avenida Josefa Ortiz De Domínguez 304, Toluca CP 50150, Mexico

**Keywords:** cell damage, gene expression, contaminants, antioxidant activity

## Abstract

The Tepetitlán reservoir is affected by untreated domestic wastewater, agricultural runoff, and livestock-related discharges, posing risks to aquatic ecosystems. This study assessed its water quality and evaluated toxic effects on adult zebrafish (*Danio rerio*). Water samples from six sites (A–F) were analyzed for physicochemical parameters and the presence of metals and pharmaceuticals, quantified at concentrations in the µg L^−1^ range. While most physicochemical parameters complied with Mexican regulations, true color exceeded established limits, and the presence of contaminants indicated environmental deterioration. Zebrafish were exposed to water from each site for 12, 24, 48, 72, and 96 h. Oxidative stress (OS) biomarkers were measured in the brain, gill, gut, and liver at all time points, and gene expression of antioxidant, detoxification, and apoptosis-related genes was assessed at 96 h in these organs. Significant OS was detected across sites, with increased lipid peroxidation, protein carbonylation, and hydroperoxide levels. Although antioxidant enzymes were activated, their response did not fully counteract oxidative damage. Gene expression analysis revealed upregulation of stress- and apoptosis-related genes. These findings demonstrate that the Tepetitlán reservoir contains pollutant mixtures capable of inducing oxidative and molecular stress responses in zebrafish, underlining potential ecological risks and the need for mitigation efforts.

## 1. Introduction

The Tepetitlán reservoir is situated within the Lerma–Chapala–Santiago basin, one of the most significant hydrological regions in Mexico, encompassing approximately 132,724 km^2^, or 7% of the national territory [1]. Located in the State of Mexico (19°54′ N, 99°31′ W), this reservoir plays a pivotal role in supplying water for both irrigation and domestic consumption across the region. Covering an area of approximately 1.82 km^2^ and with a storage capacity of around 3.4 Mm^3^, Tepetitlán is a shallow reservoir that is subjected to considerable anthropogenic pressures [2].

The Jaltepec River constitutes the primary inflow, directly contributing to the reservoir’s water volume. Additionally, seasonal drainage channels (streams) and runoff from agricultural lands and rural settlements provide further inputs, carrying nutrients, untreated domestic wastewater, and livestock-related discharges. These combined inputs significantly influence the reservoir’s chemical and biological composition [3].

The shallow depth, restricted water exchange, and prolonged hydraulic residence time—particularly during the dry season (November–May)—favor contaminant retention and accumulation [4]. Consequently, the reservoir exhibits signs of eutrophication, declining water quality, and ecological degradation, which threaten aquatic biodiversity and limit its use for fisheries, recreation, and other socioeconomic purposes [5].

Similar freshwater systems have revealed the presence of metals, pharmaceuticals, and other organic pollutants able to induce biochemical stress and compromise organismal health [6]. However, in the Tepetitlán reservoir, the ecological risks associated with such contaminants have not yet been quantified through a comprehensive ecotoxicological assessment. To address this gap, biological markers represent a valuable tool for detecting early stress responses in aquatic organisms exposed to environmental pollutants. Biomarkers related to oxidative balance, detoxification pathways, or apoptosis offer mechanistic insights into pollutant effects at cellular and tissue levels [7]. Oxidative stress (OS) is among the most widely used biomarker categories, given that excessive production of reactive oxygen species (ROS) can damage biomolecules, disrupt cellular functions, and alter physiological homeostasis [8]. In parallel, changes in gene expression reflect alterations in detoxification processes, repair mechanisms, and apoptosis, providing further mechanistic context on pollutant-induced toxicity [9].

Fish are widely recognized as effective bioindicators due to their sensitivity to environmental disturbances and ecological relevance [10]. Among them, zebrafish (*Danio rerio*) is a well-established model in toxicology. Their rapid life cycle, small size, and ease of husbandry enable controlled experimental assays, while their vertebrate physiology—featuring central nervous, immune, hepatic, renal, and circulatory systems—makes them particularly well-suited to studying molecular and physiological responses to environmental contaminants [11,12].

In this context, we evaluated contaminant-driven toxicity in the Tepetitlán reservoir by exposing adult zebrafish to environmental water samples collected from six different sites. By examining oxidative damage and gene expression profiles in multiple organs, this study provides mechanistic insight into the biological effects induced by environmental pollution in this reservoir. These findings contribute to its ecological risk assessment and support informed environmental management strategies.

## 2. Results

### 2.1. Determination of Physicochemical Parameters

**Figure 1:**
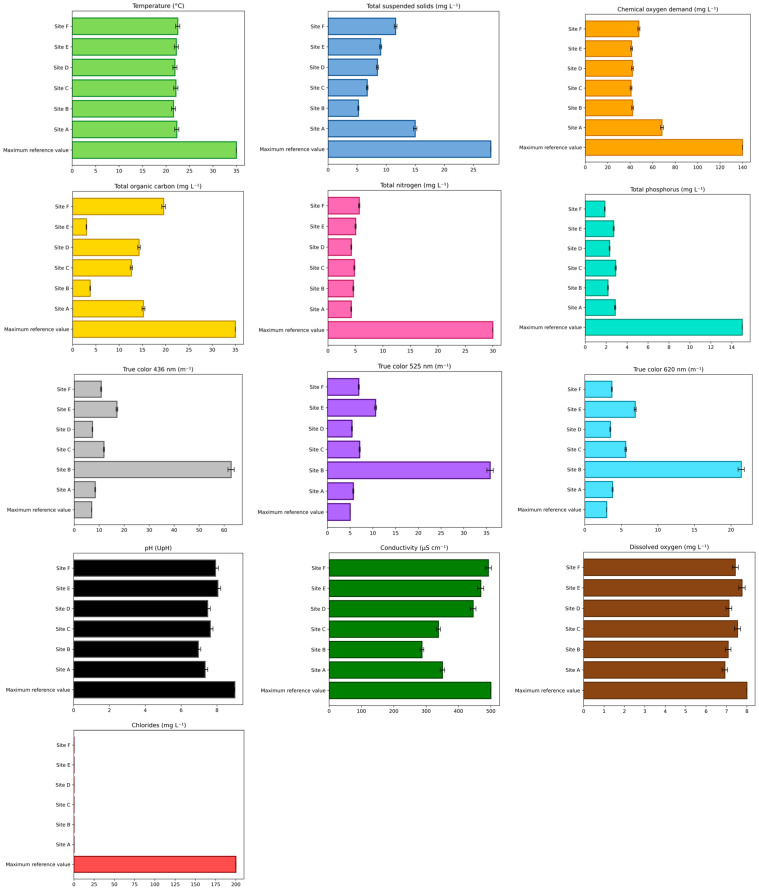
Physical and chemical parameters evaluated in the water at each sampling site of the Tepetitlán reservoir. The maximum reference values correspond to those established in NOM-001-SEMARNAT-2021. The values for the sampling sites (A–F) represent the mean of three replicates ± SD.

To evaluate the water quality of the Tepetitlán reservoir, a physicochemical analysis was performed (Figure 1) following the guidelines of the Mexican norm NOM-001-SEMARNAT-2021 [13]. Additionally, conductivity, dissolved oxygen, and chloride concentrations were measured to provide further information on the water status. Conductivity was considered acceptable between 150 and 500 µS cm^−1^ [14], dissolved oxygen within 7–8 mg L^−1^ [15], and chloride up to 200 mg L^−1^ [16]. Among all parameters, only true color exceeded the limits established by the Mexican norm at all study sites.

### 2.2. Detection and Quantification of Contaminants Present in Reservoir Water

Table 1 and Table 2 summarize the concentrations of metals and pharmaceuticals detected at the six sampling sites of the Tepetitlán reservoir, along with the maximum permissible values established by NOM-001-SEMARNAT-2021 [13]. Among the metals, arsenic, cadmium, copper, chromium, nickel, lead, and zinc were detected at several sites, whereas mercury was not quantifiable at any site. The highest concentrations of cadmium and arsenic were recorded at site C (160.0 ± 3.4 and 25.0 ± 2.2 µg L^−1^, respectively). Although all detected metals were below regulatory thresholds, some exhibited marked spatial variability, such as lead, which was detected only at sites B, C, D, and E.

Regarding pharmaceutical compounds, the predominant groups identified were antibiotics, nonsteroidal anti-inflammatory drugs (NSAIDs), and analgesics. Dicloxacillin showed a particularly high concentration at site A (469.8 ± 13.4 µg L^−1^) compared with the other sites. Sulfamethoxazole, metformin, and carbamazepine were detected only at specific sites, while dexamethasone, ketorolac, and telmisartan remained consistently below the detection limit. Among the NSAIDs, paracetamol was present at all sites, with concentrations ranging from 46.1 ± 1.3 to 75.4 ± 1.17 µg L^−1^. Naproxen was quantified at sites A, B, C, and E. In addition, the synthetic steroid 17α-ethinylestradiol was detected only at sites A, C, and E, with concentrations reaching up to 13.6 ± 0.4 µg L^−1^.

### 2.3. OS Biomarkers

**Figure 2:**
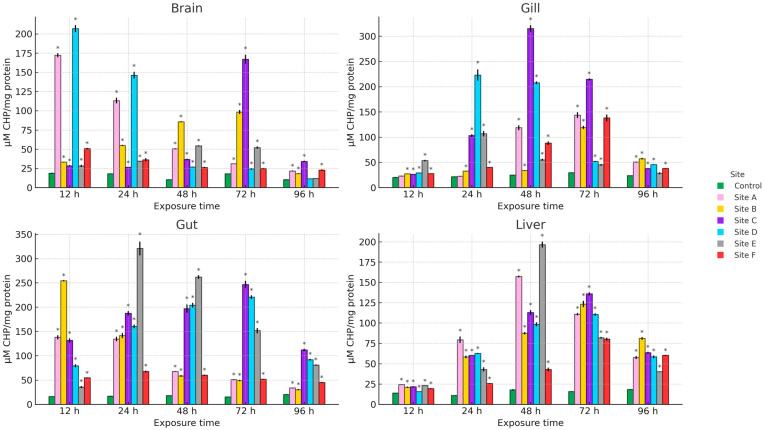
HPC in the brain, gill, gut, and liver of Danio rerio exposed for 12, 24, 48, 72, and 96 h to water from Sites A–F of the Tepetitlán reservoir. Values are expressed as mean ± SE (*n* = 3), in µM CHP/mg protein. Cumene hydroperoxide (CHP). Two-way ANOVA (factors: Time, Site, and Time × Site) was applied separately for each organ, followed by Dunnett post hoc tests with Sidak correction to compare each site with the control at the corresponding exposure time. Asterisks (*) above the bars indicate significant differences from the control group (*p* < 0.05). F-values for each factor and organ are presented in Table A1 (Appendix A.1).

**Figure 3:**
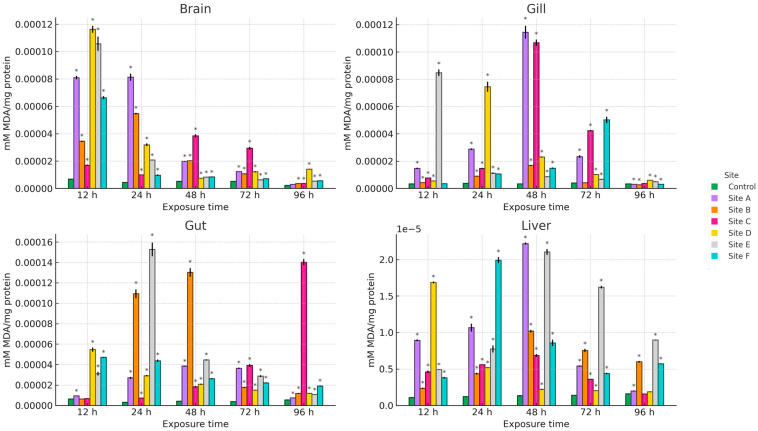
LPX levels in the brain, gill, gut, and liver of Danio rerio exposed for 12, 24, 48, 72, and 96 h to water from Sites A–F of the Tepetitlán reservoir. Values are expressed as mean ± SE (*n* = 3), in mM MDA/mg protein. Two-way ANOVA (factors: Time, Site, and Time × Site) was applied separately for each organ, followed by Dunnett post hoc tests with Sidak correction to compare each site with the control at the corresponding exposure time. Asterisks (*) above the bars indicate significant differences from the control group (*p* < 0.05). F-values for each factor and organ are presented in Table A1 (Appendix A.1).

**Figure 4:**
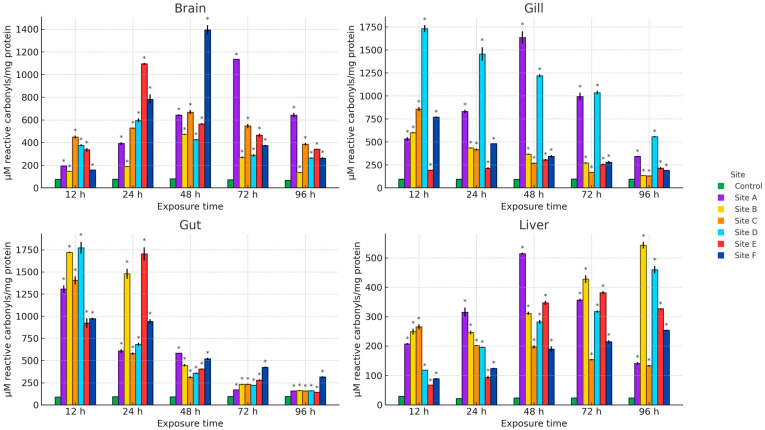
PCC in the brain, gill, gut, and liver of *Danio rerio* exposed for 12, 24, 48, 72, and 96 h to water from Sites A–F of the Tepetitlán reservoir. Values are expressed as mean ± SE (*n* = 3), in μM reactive carbonyls/mg protein. Two-way ANOVA (factors: Time, Site, and Time × Site) was applied separately for each organ, followed by Dunnett post hoc tests with Sidak correction to compare each site with the control at the corresponding exposure time. Asterisks (*) above the bars indicate significant differences from the control group (*p* < 0.05). F-values for each factor and organ are presented in Table A1 (Appendix A.1).

Figure 2, Figure 3 and Figure 4 present the results of hydroperoxide content (HPC), lipid peroxidation (LPX), and protein carbonyl content (PCC). An increase in these biomarkers relative to the control group was observed across all study sites, organs, and exposure times. For HPC, the highest increase in brain occurred at site D at 12 h (1008.4%), in gill at site C at 48 h (1164.5%), and in gut and liver at site E at 24 h (1759.1%) and 48 h (1033%), respectively. Regarding LPX, the maximum increase in brain was recorded at site D at 12 h (1604.8%), whereas in gill and liver the greatest values were observed at site A at 48 h (3237.8% and 1532.5%, respectively). In the gut, the maximum response was detected at site E at 24 h (4572.8%). For PCC, the highest increase in the brain was observed at site F at 48 h (1692.2%), in the gill at site D at 12 h (1718.1%), and in the gut and liver at site B at 12 h (1859.1%) and 96 h (2182.7%), respectively.

**Figure 5:**
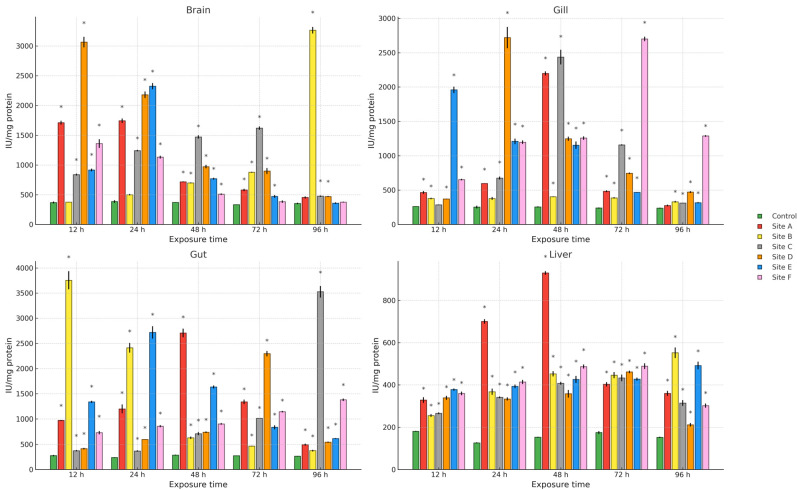
SOD activity in the brain, gill, gut, and liver of *Danio rerio* exposed for 12, 24, 48, 72, and 96 h to water from Sites A–F of the Tepetitlán reservoir. Values are expressed as mean ± SE (*n* = 3), in IU/mg protein. International Units (IU). Two-way ANOVA (factors: Time, Site, and Time × Site) was applied separately for each organ, followed by Dunnett post hoc tests with Sidak correction to compare each site with the control at the corresponding exposure time. Asterisks (*) above the bars indicate significant differences from the control group (*p* < 0.05). F-values for each factor and organ are presented in Table A1 (Appendix A.1).

**Figure 6:**
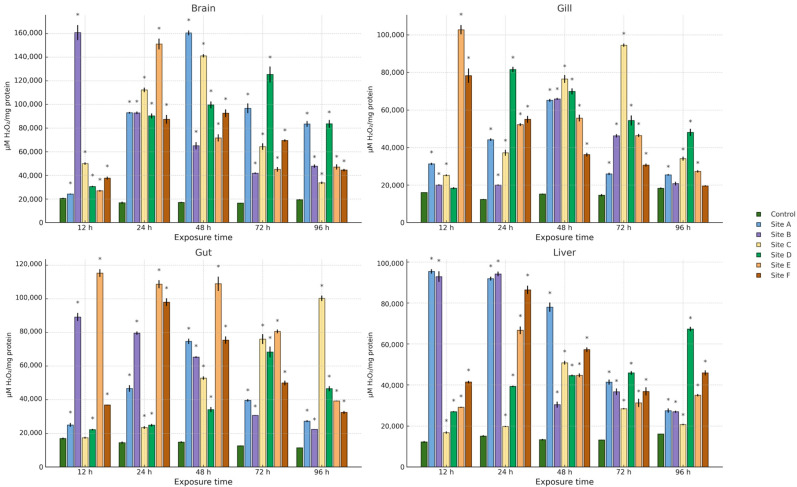
CAT activity in the brain, gill, gut, and liver of *Danio rerio* exposed for 12, 24, 48, 72, and 96 h to water from Sites A–F of the Tepetitlán reservoir. Values are expressed as mean ± SE (*n* = 3), in μM H_2_O_2_/mg protein. Two-way ANOVA (factors: Time, Site, and Time × Site) was applied separately for each organ, followed by Dunnett post hoc tests with Sidak correction to compare each site with the control at the corresponding exposure time. Asterisks (*) above the bars indicate significant differences from the control group (*p* < 0.05). F-values for each factor and organ are presented in Table A1 (Appendix A.1).

Regarding the activity of the antioxidant enzymes superoxide dismutase (SOD) and catalase (CAT), an increase relative to the control group was observed across all study sites, organs, and exposure times (Figure 5 and Figure 6). For SOD, the greatest increase in the brain was recorded at site B at 96 h (770.1%), in the gill at site D at 24 h (965.8%), in the gut at site B at 12 h (1245.3%), and in the liver at site A at 48 h (500.6%). For CAT, the maximum increase in brain was observed at site B at 12 h (776.2%), while in gill and gut it occurred at site E at 12 h (531.6% and 567.2%, respectively), and in liver at site A at 12 h (674.5%).

### 2.4. Gene Expression

**Figure 7:**
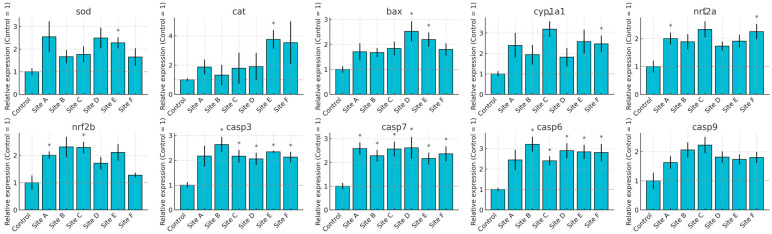
Relative mRNA expression of oxidative stress, biotransformation, and apoptosis-related genes in the brain of *Danio rerio* exposed to water from Sites A–F of the Tepetitlán reservoir. Values are expressed as mean ± SE (*n* = 6), normalized to the control group (control = 1). The dashed line indicates the control level. Asterisks (*) above the bars indicate significant differences from the control group (*p* < 0.05, Sidak-corrected *t*-test). Corresponding ANOVA F values are provided in Table A2 (Appendix A.2).

**Figure 8:**
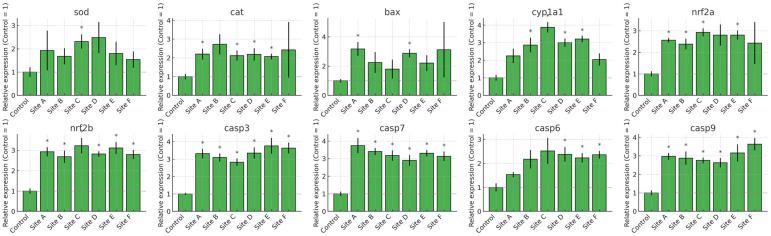
Relative mRNA expression of oxidative stress, biotransformation, and apoptosis-related genes in the gill of *Danio rerio* exposed to water from Sites A–F of the Tepetitlán reservoir. Values are expressed as mean ± SE (*n* = 6), normalized to the control group (control = 1). The dashed line indicates the control level. Asterisks (*) above the bars indicate significant differences from the control group (*p* < 0.05, Sidak-corrected *t*-test). Corresponding ANOVA F values are provided in Table A2 (Appendix A.2).

**Figure 9:**
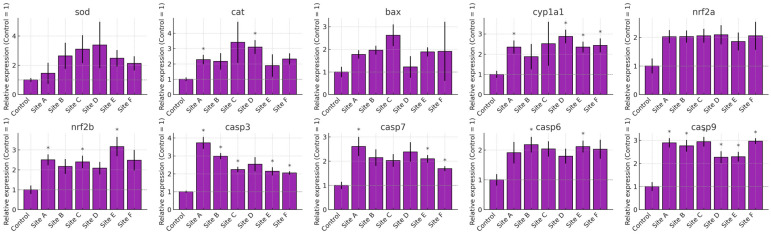
Relative mRNA expression of oxidative stress, biotransformation, and apoptosis-related genes in the gut of *Danio rerio* exposed to water from Sites A–F of the Tepetitlán reservoir. Values are expressed as mean ± SE (*n* = 6), normalized to the control group (control = 1). The dashed line indicates the control level. Asterisks (*) above the bars indicate significant differences from the control group (*p <* 0.05, Sidak-corrected *t*-test). Corresponding ANOVA F values are provided in Table A2 (Appendix A.2).

**Figure 10:**
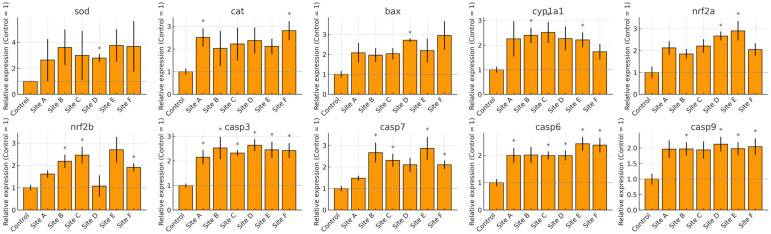
Relative mRNA expression of oxidative stress, biotransformation, and apoptosis-related genes in the liver of *Danio rerio* exposed to water from Sites A–F of the Tepetitlán reservoir. Values are expressed as mean ± SE (*n* = 6), normalized to the control group (control = 1). The dashed line indicates the control level. Asterisks (*) above the bars indicate significant differences from the control group (*p* < 0.05, Sidak-corrected *t*-test). Corresponding ANOVA F values are provided in Table A2 (Appendix A.2).

The transcriptional profiles of *Danio rerio* exposed to water from Sites A–F of the Tepetitlán reservoir revealed marked, organ-specific alterations in genes associated with oxidative stress, detoxification, and apoptosis (Figure 7, Figure 8, Figure 9 and Figure 10). In the brain, significant upregulation of *sod*, *cat*, and *nrf2a* occurred mainly at Sites C–E, while *bax*, *casp7*, and *casp9* showed significant increases at several locations, indicating activation of antioxidant and apoptotic pathways. Gills exhibited the strongest and most widespread responses. *sod*, *cat*, *nrf2a*, and *nrf2b* were significantly upregulated at multiple sites, particularly Sites A and C. Apoptotic markers—including *bax*, *casp3*, *casp6*, *casp7*, and *casp9*—also showed broad induction. Additionally, *cyp1a1* increased markedly, reflecting the high sensitivity of gill tissue to waterborne contaminants. In the gut, gene expression changes were moderate but still site-dependent. Significant induction of *sod*, *cat*, *nrf2a*, *bax*, and multiple caspases was observed at specific sites. These patterns indicate localized oxidative and apoptotic responses. The liver displayed a broad activation pattern. *sod*, *cat*, *nrf2a*, and *nrf2b* were significantly elevated at Sites B–E. Expression of *bax*, *casp3*, *casp7*, and *casp9* also increased across multiple sites. In addition, *cyp1a1* was significantly upregulated at Sites B and C. Together, these responses confirm hepatic involvement in antioxidant defense, apoptosis, and biotransformation.

## 3. Discussion

The characterization of a water body is fundamental for understanding its current state, evaluating its quality, and predicting future trends. This process enables the identification of contaminants and their potential sources, as well as the analysis of physicochemical, biological, and microbiological parameters that indicate water quality [17,18]. Physicochemical evaluation is a key component of water quality assessment, as it provides information on parameters such as temperature, pH, conductivity, turbidity, hardness, and dissolved oxygen. Nevertheless, this type of analysis has limitations, particularly its inability to detect organic and inorganic contaminants at trace concentrations, which necessitates the use of advanced analytical techniques for detection and quantification [19,20]. In this study, all physicochemical parameters measured were within Mexican regulatory limits and the reference values for conductivity, dissolved oxygen, and chlorides, except for true color. At all study sites, true color values exceeded the limits established by NOM-001-SEMARNAT-2021 [13]. This parameter is a relevant indicator of water quality because it reflects the presence of dissolved organic and inorganic substances. It is also considered a more precise measure of contamination by humic and fulvic acids, metals, and other compounds derived from natural or anthropogenic sources. These substances can alter the taste, odor, and toxicity of water due to the formation of more bioavailable complexes capable of inducing OS and affecting essential physiological functions in aquatic organisms [21]. Moreover, they can modify physicochemical parameters such as pH, conductivity, and chemical oxygen demand, thereby exacerbating their toxic effects. The consistently elevated true color observed across all sites suggests widespread organic contamination, likely associated with untreated domestic effluents, agricultural runoff, and livestock activities.

Additionally, metals (µg L^−1^) such as arsenic, cadmium, chromium, copper, nickel, lead, and zinc were detected and quantified, along with pharmaceuticals (µg L^−1^) including dicloxacillin, sulfamethoxazole, metformin, carbamazepine, diclofenac, naproxen, paracetamol, and 17α-ethinylestradiol. Sources of water contamination by heavy metals and pharmaceuticals include landfill leachates, municipal and industrial wastewater, the indiscriminate disposal of domestic and hospital waste, and urban runoff. In the case of metals, natural processes such as erosion and mining residues also contribute to their presence in aquatic systems [22,23,24]. These pollution sources have been identified in the Tepetitlán reservoir. Although typical concentrations of pharmaceuticals in surface waters typically fall within the ng L^−1^ to low µg L^−1^ range, several studies have reported substantially higher levels in water bodies heavily impacted by untreated domestic or industrial effluents. For example, 17α-ethinylestradiol has been detected at 624.3 µg L^−1^ in surface water from the Apatlaco River in Mexico [25]. Similarly, diclofenac concentrations up to 107.87 µg L^−1^ have been reported in surface waters from the Owabi and Barekese reservoirs in Ghana [26], and naproxen (59.3 µg L^−1^) and diclofenac (1.01–10.2 µg L^−1^) have been measured in the Umgeni River in South Africa [27]. In Brazil, surface water from the Beberibe River contained diclofenac at 193 µg L^−1^ and paracetamol at 42 µg L^−1^ [28]. Comparable ranges have also been documented for heavy metals. In the Villa Victoria Dam (Mexico), surface water samples contained metals at concentrations ranging from 0.3 to 740 µg L^−1^ [29], demonstrating that elevated metal levels can occur in reservoirs influenced by municipal effluents and watershed activities.

The presence of pharmaceuticals and heavy metals in aquatic systems adversely affects water quality, ecosystems, and human health. These pollutants can bioaccumulate in aquatic organisms, leading to an imbalance in the production of ROS, highly reactive molecules that damage lipids, proteins, and DNA [30]. OS, defined as the disruption of the balance between ROS generation and antioxidant defenses, is widely used as a non-specific biomarker of toxicity in multipollutant environments, since diverse contaminants can trigger this imbalance [31]. In this study, the brain was selected because of its high vulnerability to oxidative damage due to its elevated oxygen consumption, high content of polyunsaturated fatty acids, and relatively low antioxidant capacity compared with other organs [32]. The gills, which play a central role in gas exchange, also represent a major entry route for toxicants such as heavy metals and pharmaceuticals, making them highly sensitive to pollutants and environmental changes [33]. The gut was considered because it acts as a first barrier against ingested substances [34], while the liver was included given its central role in xenobiotic metabolism and detoxification [35].

Drugs can generate ROS either as part of their mechanism of action or as a side effect of their metabolism [36]. Heavy metals are also capable of inducing ROS formation through redox reactions [37]. LPX, a key oxidative process, begins when ROS attack polyunsaturated fatty acids in cell membranes, leading to the formation of lipid radicals. These radicals react with oxygen to produce lipid hydroperoxides as the primary products of peroxidation [38]. However, these hydroperoxides are unstable and, if not neutralized by antioxidants, decompose into secondary products such as aldehydes (e.g., malondialdehyde) and other reactive compounds that can damage biomolecules [39]. In this study, significant increases in HPC and LPX were observed in all organs analyzed at different exposure times. Proteins are also highly susceptible to ROS, as hydroxyl radicals (•OH), hydrogen peroxide (H_2_O_2_), and superoxide anions (O_2_•^−^) can interact with amino acid side chains, causing oxidative modifications [40]. The most vulnerable amino acids are those containing sulfhydryl (-SH) groups, such as cysteine, and those with aromatic rings, such as tyrosine and tryptophan, which can undergo oxidation leading to cross-linking, carbonyl formation, and other modifications [41]. Such alterations can disrupt protein structure and function, resulting in loss of enzymatic activity, aggregation, and even cell death [42]. In this work, significant increases in PCC were detected in all organs across the different exposure times.

Antioxidant defenses are essential to counteract oxidative damage. The antioxidant enzymes SOD and CAT play a central role in neutralizing ROS. SOD catalyzes the dismutation of O_2_•^−^ into H_2_O_2_ and O_2_ [43], while CAT subsequently converts H_2_O_2_ into H_2_O and O_2_, preventing the formation of highly damaging •OH radicals [44]. Under moderate OS, SOD and CAT activity typically increase as an adaptive response to neutralize excess ROS. However, under sustained or severe OS, excessive ROS can inactivate or deplete these enzymes, impairing their protective function [45]. Continuous exposure to high ROS levels may also induce structural damage to SOD and CAT, further reducing their effectiveness [46]. In this study, both enzymes showed significant increases in activity in all organs, followed by a decline after reaching peak levels. This pattern suggests that, during the early stages of exposure, antioxidant enzymes effectively counteracted ROS, as reflected by decreased oxidative damage. Nevertheless, prolonged exposure overwhelmed antioxidant defenses, leading to reduced enzymatic activity and increased oxidative damage at later time points.

The oxidative stress observed in this study on zebrafish is a consequence of the presence of contaminants in the Tepetitlán reservoir. This finding is consistent with other studies that evaluated the toxicity of metals. For example, Dai et al. demonstrated that cadmium at concentrations of 250 and 500 μg L^−1^ induces OS in the liver of *Procypris merus*. The authors reported an increase in MDA and a reduction in the enzymatic activity of SOD, CAT, and glutathione S-transferase (GST) after 30 days of exposure [47]. Similarly, Zhao et al. reported lead accumulation in the gills, gut, liver, and muscles of *Channa argus*. Exposure to concentrations of 50,000, 200,000, and 800,000 μg L^−1^ resulted in a significant decrease in CAT and glutathione peroxidase (GPx) in the liver and gill, along with an increase in MDA and PCC after 14 and 28 days [48]. Shaw et al. demonstrated that, at an environmentally relevant concentration (2000 μg L^−1^), chromium induced an increase in MDA and reduced glutathione (GSH) and CAT activity in the liver and gill at exposure times of 1, 7, 15, 30, and 60 days [49]. Likewise, Kumar et al. reported an increase in SOD, CAT, and GST in the liver, gill, brain, and kidney of *Pangasianodon hypophthalmus* after 96 h of arsenic exposure at concentrations of 25,000–30,000 μg L^−1^ [50]. In addition, Gopi et al. showed that, at both low (40 μg L^−1^) and high (400 μg L^−1^) copper concentrations, there was accumulation in liver and gill tissues of *Oreochromis niloticus*. The authors also observed a reduction in antioxidant enzymes (SOD, CAT, GPx, and GST), along with an increase in MDA and PCC, after 30 and 60 days of exposure [51]. Furthermore, Shahzad et al. reported that, at a concentration of 1500 μg L^−1^, there was maximum zinc accumulation (3064.3 μg Kg^−1^) in the liver of *Oreochromis mossambicus*. They also observed a greater increase in LPX, CAT, and GSH in the gill compared to the liver, whereas SOD activity was higher in the liver [52].

Regarding studies reported for some drugs detected and quantified in this work, Taher et al. concluded that metformin induces cellular oxidative stress by reducing SOD activity and total antioxidant capacity (TAC) in *Clarias gariepinus* after exposure to 50,000 μg L^−1^ for 7 days [53]. Similarly, Gasca-Pérez et al. demonstrated that carbamazepine at 2000 μg L^−1^ causes oxidative damage in the liver, gill, and brain of *Cyprinus carpio*, with increases in HPC, LPX, and PCC, along with decreases in the antioxidant enzymes SOD, CAT, and GPx [54]. In addition, Gutiérrez-Noya et al. reported that paracetamol induces oxidative damage in *Cyprinus carpio* larvae at concentrations ranging from 0.5 to 3.5 μg L^−1^. The authors observed increases in HPC, LPX, and the enzymatic activity of SOD and CAT [55]. Furthermore, Priyadarshinee et al. concluded that naproxen at concentrations of 1 and 50 μg L^−1^ generates oxidative stress in *Labeo rohita*. They evaluated exposure for 35 days at 7-day intervals and showed a time- and concentration-dependent increase in ROS, LPX, and GST, along with a time- and concentration-dependent decrease in SOD, CAT, GPx, and glutathione [56].

Finally, ROS can damage DNA and modify proteins essential for transcriptional regulation. These molecular changes trigger a cascade of events that may alter the expression of a wide range of genes, including those involved in stress response, DNA repair, and apoptosis [57]. In this study, genes involved in different responses to OS were evaluated. The *sod* and *cat* genes, essential components of the antioxidant defense system, encode the enzymes SOD and CAT, respectively, which neutralize ROS generated by metals and pharmaceuticals, thereby protecting cells from oxidative damage [58]. Likewise, *nfe2l2a* and *nfe2l2b* regulate the antioxidant response by activating the expression of genes that counteract OS [59]. The *cyp1a* gene encodes a cytochrome P450 enzyme that metabolizes xenobiotics, including certain metals and drugs, and generates ROS during this process, contributing to OS [60]. At the same time, the genes *baxa*, *casp3*, *casp6*, *casp7*, and *casp9* are associated with apoptosis and play a key role when cellular damage is irreversible. *Baxa* promotes mitochondrial permeabilization, whereas caspases (*casp3*, *casp6*, *casp7*, and *casp9*) are proteases that orchestrate the apoptotic cascade, driving programmed cell death in response to excessive OS [61]. The regulation and activation of these genes under exposure to metals and pharmaceuticals are essential to maintain the balance between cell protection and elimination of damaged cells, thus contributing to organismal homeostasis. In this study, the expression of these genes was observed across all sampling sites, indicating that organisms are responding to OS induced by contaminants in the Tepetitlán reservoir. However, this protective response appears to be exceeded, as cells are progressing toward apoptosis.

This behavior is consistent with previous studies. For example, Wang et al. reported increased expression of apoptosis-related genes (*p53*, *casp3*, *casp9*, and *bax*), along with decreased *bcl2* expression, in the liver of *Takifugu fasciatus* after exposure to copper (20 and 100 μg L^−1^) for 30 days [62]. Similarly, Shaw et al. demonstrated transcriptional induction of apoptotic genes (*bax*, *casp9*, *casp3*) and down-regulation of *bcl2* in the brain of *Danio rerio* exposed to chromium (2000 μg L^−1^) [49]. Dai et al. found that cadmium exposure (250 and 500 μg L^−1^) reduced the transcriptional expression of antioxidant-related genes (*gpx1*, *gpx2*, *cata*, *gsta1*, *sod1*, and *nrf2*) in the liver of *Procypris merus* [47]. Furthermore, Bao et al. demonstrated that *nrf2* expression was inhibited at 24 h but induced at 168 h following exposure to diclofenac (1.572 × 10^−3^, 1.572 × 10^−2^, 0.1572, and 1.572 μmol L^−1^) in *Gambusia affinis* [63]. In addition, Paravani et al. reported increased mRNA levels of *sod* and *cat* in the gill of *Danio rerio* after exposure to 0.005 μg L^−1^ of 17α-ethinylestradiol [64].

## 4. Materials and Methods

### 4.1. Sampling of Water from the Tepetitlán Reservoir

**Figure 11:**
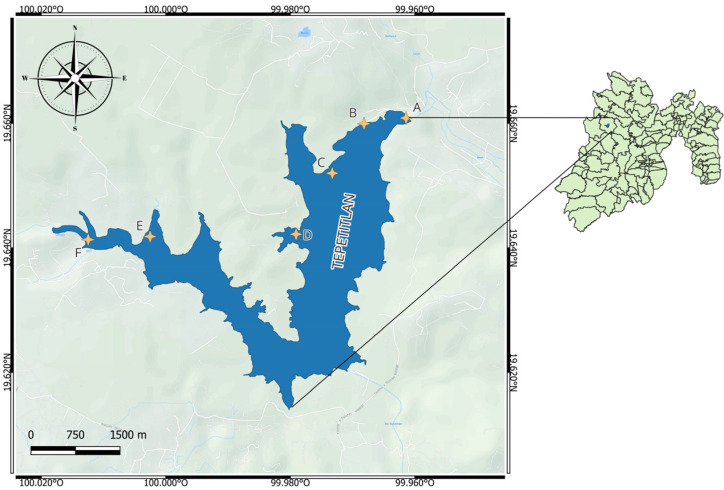
Location of sampling sites at Tepetitlán reservoir, State of Mexico (image processed using QGIS 3.38 software).

Figure 11 depicts the geographical distribution of the sampling stations throughout the Tepetitlán reservoir. The selected locations encompass diverse environmental influences: Sites E and F are mainly affected by domestic effluents, livestock operations, and washing activities; Site D corresponds to the pumping system of the Estutempan water treatment facility; Site C represents an area dominated by agricultural inputs; Site B receives mixed discharges from urban and farming sources; and Site A is positioned at the reservoir’s main gate. Sampling sites were selected based on dominant contamination sources rather than geographical symmetry; opposite riverbanks did not exhibit independent pollution inputs or land-use differences and therefore were not expected to provide additional relevant environmental variation.

Water samples of 40 L were obtained from each station in compliance with the Mexican regulation NMX-AA-003-1980 [65]. Sampling was performed in January 2024 at a depth of 1 m. In the State of Mexico, January corresponds to the dry season, which typically extends from November to May and is characterized by minimal rainfall and reduced surface runoff. Conducting sampling during this period minimizes hydrological variability and allows for a clearer assessment of contaminant concentrations associated with constant point and non-point sources, without the dilution effects typical of the rainy season.

Immediately after collection, water samples were transferred into polyethylene containers, stored at 4 °C, protected from light, and transported to the Environmental Toxicology Laboratory of the Faculty of Chemistry at the Autonomous University of the State of Mexico. Samples for toxicological and physicochemical evaluations were processed within 24 h, while those intended for metal and pharmaceutical analyses were preserved under the same conditions and processed within 5 days.

### 4.2. Physicochemical Characterization of Water

The physicochemical parameters of the water were assessed following the criteria outlined in the Mexican regulation NOM-001-SEMARNAT-2021 [13], which establishes permissible concentrations of pollutants in national water bodies. In this study, the norm was used solely as a regulatory point of reference to contextualize the measurements, rather than to evaluate compliance or ecological risk in natural systems.

The parameters analyzed included temperature, pH, dissolved oxygen (DO), conductivity, true color (436, 525, and 620 nm), chlorides, total suspended solids (TSS), total nitrogen (TN), total organic carbon (TOC), total phosphorus (TP), and chemical oxygen demand (COD). All analyses were performed in triplicate. Quality Assurance/Quality Control (QA/QC) included procedural blanks, calibration verification every 10 samples, and acceptance of replicate variability only when <5%. All instruments and reagents complied with NOM-001 methodological requirements.

Temperature, pH, dissolved oxygen, and electrical conductivity were measured in situ at each sampling site using a calibrated multiparameter probe (HANNA HI9829, Hanna Instruments, Woonsocket, RI, USA). Calibration was performed before each sampling campaign using standard buffer solutions for pH (4.01, 7.00, and 10.01), a 1413 µS cm^−1^ conductivity standard, and air-saturated water for DO calibration.

True color was determined at 436, 525, and 620 nm following the spectrophotometric method described in NOM-001. Samples were filtered through 0.45 µm membranes before measurement and analyzed using a UV–Vis spectrophotometer (Hach DR6000, Hach Company, Loveland, CO, USA). Calibration curves were prepared using platinum–cobalt color standards.

TSS were quantified in accordance with NOM-001. 1 L of water was filtered through pre-weighed GF/C glass fiber filters (Whatman, Maidstone, UK). Filters were dried at 103–105 °C for 1 h, cooled in a desiccator, and reweighed to determine suspended solids concentration.

COD was measured using the closed reflux, colorimetric method specified in NOM-001 (Hach Method 8000; Hach Company, Loveland, CO, USA). High-range dichromate digestion vials (0–1500 mg L^−1^) were used. Samples were digested at 150 °C for 2 h, and absorbance was read at 620 nm.

TN and TPwere analyzed following the digestion and colorimetric methods of NOM-001. TN was determined using alkaline persulfate digestion followed by UV absorbance detection. TP was quantified using the ascorbic acid–molybdate reaction after acid digestion, measuring absorbance at 880 nm.

TOC was determined using high-temperature catalytic combustion in a TOC analyzer (TOC-L; Shimadzu Corporation, Kyoto, Japan), in accordance with NOM-001. Calibration curves were prepared using potassium hydrogen phthalate standards.

Chloride concentration was analyzed following the argentometric method in NOM-001. Water samples were titrated with standardized silver nitrate solution using potassium chromate as an indicator.

### 4.3. Determination of Organic and Inorganic Contaminants in Water

#### 4.3.1. Pharmaceutical Analysis Through SPE–HPLC-UV

Water samples collected at each site (500 mL) were used for the determination of pharmaceutical residues. Immediately after collection, the samples were vacuum-filtered through Whatman GF/C glass fiber filters (1.2 μm) to remove suspended solids. The clarified water was then subjected to solid-phase extraction using Sep-Pak^®^ C18 cartridges (1 g; Waters Corporation, Milford, MA, USA), previously conditioned with chromatographic-grade methanol followed by Milli-Q water. The samples were loaded at a flow rate of 2–3 mL min^−1^, after which the cartridges were dried under vacuum and the retained analytes were eluted with methanol. The eluates were evaporated to dryness and reconstituted in 1 mL of Milli-Q water. Method performance was evaluated through recovery assays in fortified environmental matrices (250–1000 μg L^−1^). Recoveries ranged between 82.67% and 114.44%, with relative deviations below 15%, and these values were applied as correction factors during quantification. Chromatographic analyses were performed using HPLC with UV detection and an Eclipse XDB-C18 column (4.6 × 250 mm, 5 μm; Agilent Technologies, Santa Clara, CA, USA). Calibration curves prepared in ultrapure water covered a concentration range of 0.1–10,000 μg L^−1^ and showed strong linearity (R^2^ > 0.99). Blanks remained below the detection limit throughout the analytical sequence, and fortified samples were included for quality control. The target pharmaceuticals included dicloxacillin (DCX), trimethoprim (TMP), sulfamethoxazole (SMX), 17α-ethinylestradiol (EE2), metformin (MET), carbamazepine (CBZ), dexamethasone (DEX), diclofenac (DCF), naproxen (NPX), paracetamol (PCT), ketorolac (KET), and telmisartan (TLM). Chromatographic conditions were tailored to each compound. A flow rate of 1 mL min^−1^ was used for DCX, MET, CBZ, KET, TLM, and PCT, whereas DCF and NPX were analyzed at 0.5 mL min^−1^. Mobile phases were selected according to analyte characteristics: DCX was separated with acetonitrile and 0.1% formic acid (70:30, *v*/*v*); MET with methanol and 1% formic acid (60:40, *v*/*v*); CBZ with methanol and 1% acetic acid (80:20, *v*/*v*); and PCT with water and methanol (60:40, *v*/*v*). The remaining compounds were analyzed with solvent systems previously validated for UV detection. Detection was performed at the wavelength corresponding to maximum absorbance for each analyte: 225 nm (DCX), 280 nm (TMP and EE2), 268 nm (SMX), 232 nm (MET), 280 nm (CBZ), 254 nm (DEX), 274 nm (DCF), 230 nm (NPX), 322 nm (KET), 300 nm (TLM), and 240 nm (PCT). The method achieved LOD values ranging from 0.0001 to 1 mg L^−1^ and LOQ values between 0.01 and 10 mg L^−1^, depending on the compound [66].

#### 4.3.2. Metal Analysis Through Acid Digestion and Spectrometric Techniques

Water samples collected from the Tepetitlán reservoir were first passed through 0.45 µm membrane filters (Merck Millipore, Burlington, MA, USA) and immediately acidified to pH < 2 with ultrapure nitric acid (Merck, Darmstadt, Germany) to preserve the dissolved metal fraction. The determination of arsenic (As), cadmium (Cd), chromium (Cr), copper (Cu), nickel (Ni), lead (Pb), and zinc (Zn) was carried out using an inductively coupled plasma optical emission spectrometer (ICP-OES; iCAP 7400 Duo; Thermo Fisher Scientific, Waltham, MA, USA). Instrumental settings included a radio-frequency generator operating at 27.12 MHz, a plasma power of 1150 W, and argon flows of 12 L min^−1^ for the plasma, 0.5 L min^−1^ for the auxiliary line, and 0.7 L min^−1^ for the nebulizer. A Meinhard concentric nebulizer coupled to a cyclonic spray chamber was used throughout the analysis. Axial plasma viewing was selected to enhance detection sensitivity, and emission lines specific to each element were used to minimize potential interferences. Quantification was performed by external calibration using certified multielement solutions, achieving correlation coefficients above 0.99. Mercury (Hg), which cannot be reliably measured under ICP-OES conditions, was analyzed separately by flame atomic absorption spectrophotometry (AAS; iCE 3000; Thermo Fisher Scientific, Waltham, MA, USA) system equipped with deuterium background correction. Analytical parameters included a lamp current of 4 mA and an air–acetylene flame with flow rates of 10.0 and 1.0 L min^−1^, respectively. Each aliquot was measured three times, and the mean value was used for reporting. Quality control procedures included the use of reagent blanks and certified control standards during each analytical sequence to verify instrument performance. The limits of detection and quantification were established from the standard deviation of blank measurements and the slopes of the calibration curves. Depending on the analyte, LOD values ranged from 0.1 to 1.7 μg L^−1^ and LOQ values from 0.3 to 3.0 μg L^−1^. The emission wavelengths used for As, Cd, Cr, Cu, Ni, Pb, and Zn were 193.696, 228.802, 205.560, 324.700, 241.476, 221.418, and 213.856 nm, respectively. To evaluate method accuracy, recovery tests were conducted by spiking environmental samples with known concentrations of each metal. Recoveries were consistently high, falling between 89.5% and 102.1%, with relative standard deviations below 8%. Specifically, the observed recoveries were 90.4 ± 7.2% for As, 101.3 ± 7.7% for Cd, 96.9 ± 7.8% for Cu, 94.6 ± 7.5% for Cr, 89.5 ± 6.5% for Pb, and 102.1 ± 7.9% for Zn [66].

### 4.4. Ethical Approval, Animal Care, and Exposure Design

All experimental procedures were carried out in accordance with national and institutional ethical regulations for animal research. The study protocol was reviewed and approved by the Ethics and Research Committee of the Autonomous University of the State of Mexico (UAEM) under approval number RP.UAEM.ERC.132.2020. All procedures complied with the Mexican Official Norm NOM-062-ZOO-1999, which governs the care and use of laboratory animals [67].

Adult wild-type zebrafish (*Danio rerio*) were used throughout the study. Fish were 4 months old upon arrival and approximately 6 months old at the start of the experiments, with an average mass of 0.84 ± 0.07 g and a standard length of 3.48 ± 0.14 cm. All animals were obtained from a certified aquaculture facility (MX Aquanimals, Toluca, Mexico) and acclimated for two months in 120 L tanks maintained at 28 ± 1 °C under a 12 h light/12 h dark cycle. Fish were fed a commercial dry diet (TetraMin^®^ PRO; Tetra GmbH, Melle, Germany) three times daily. Adult specimens were intentionally selected because the objective of this study was to evaluate multi-organ biochemical and transcriptional responses under environmentally relevant exposure conditions rather than to conduct an OECD-standardized toxicity test, which typically requires juvenile fish.

Environmental exposure assays were conducted using 6 L tanks filled with undiluted water collected from six sampling sites (A–F) of the Tepetitlán reservoir, while the control group was maintained in clean water. All systems were kept under constant aeration at 28 ± 1 °C with a 12 h light/12 h dark photoperiod. To preserve the chemical characteristics of the environmental matrix, no filtration, chemical treatment, or water replacement was performed during the exposure period. Each treatment consisted of three independent 6 L experimental units, each containing thirty fish at a 1:1 sex ratio. Both sexes were exposed together under identical conditions to maintain a balanced representation of sex-related biological variability.

Because this study represents an environmental exposure bioassay rather than an OECD-compliant acute toxicity test, fish density did not follow OECD Guidelines 203 or 210. To ensure that density did not influence physiological endpoints, water quality parameters (pH, dissolved oxygen, ammonia, nitrite, nitrate, and conductivity) were continuously monitored and maintained within optimal ranges. In the control group, recorded values were pH 7.4 ± 0.2, dissolved oxygen 9.2 ± 0.4 mg L^−1^, nitrite 0.022 ± 0.004 mg L^−1^, nitrate 2.4 ± 0.3 mg L^−1^, un-ionized ammonia 0.007 ± 0.002 mg L^−1^, and conductivity 353 ± 22 µS cm^−1^. No abnormal behaviors, density-related stress responses, or fish mortality were observed at any time during the acclimation or exposure periods.

At each exposure interval (12, 24, 48, 72, and 96 h), six randomly selected fish per replicate were euthanized by hypothermic shock, maintaining the 1:1 sex ratio. Fish were gradually immersed in water cooled to 2–4 °C until the complete loss of opercular movements and reflex responses was confirmed. Immediately after death, the brain, gill, gut, and liver tissues were dissected for OS-related biochemical analyses. For gene-expression assays, fish were exposed under the same experimental design for 96 h. The selection of exposure times was based on the distinct temporal dynamics of the biological responses being measured. OS biomarkers were evaluated at 12, 24, 48, 72, and 96 h to capture both early and progressive biochemical alterations resulting from contaminant exposure. In contrast, gene expression analysis was conducted only at 96 h, as transcriptional changes typically require longer exposure durations to manifest and stabilize, reflecting cumulative molecular responses rather than immediate acute effects.

To reduce biological and technical variability, all experimental procedures were carefully standardized. Fish of similar age and size were used under controlled acclimation and exposure conditions, maintaining a strict 1:1 sex ratio. Euthanasia, tissue collection, and all biochemical and molecular assays were performed following uniform protocols and with the same operator and equipment, ensuring consistent and reliable measurements.

### 4.5. Assessment of the Oxidative Response

Tissues were pooled by organ and homogenized in phosphate buffer (pH 7.4). The resulting homogenates were divided into two portions (tubes 1 and 2). For tube 1, 300 μL of 20% trichloroacetic acid was added to 300 μL of homogenate. Tube 2 contained only 700 μL of homogenate. Both tubes were centrifuged at 4 °C for 15 min at 12,000× *g* and 13,800× *g*, respectively. The assessment included HPC, LPX, and PCC, along with the enzymatic activity of SOD and CAT. All measurements were conducted using the supernatant.

#### 4.5.1. HPC

The level of hydroperoxides was assessed following the procedure described by Jiang et al. Deproteinized samples (100 μL) were combined with 900 μL of an iron–xylenol orange working solution prepared in methanol. After incubating the mixture at ambient temperature for 1 h, absorbance was measured at 560 nm. Concentrations were estimated from a calibration curve and expressed as µM CHP per milligram of protein [68].

#### 4.5.2. LPX

Lipid peroxidation was evaluated using the method of Buege and Aust. Supernatants were brought to a final volume of 1 mL with Tris–HCl buffer (pH 7.4) and incubated at 37 °C for 30 min. A thiobarbituric acid/trichloroacetic acid reagent was then added, and samples were vortexed and heated for 45 min. After cooling and centrifugation, absorbance was recorded at 535 nm. MDA concentrations were calculated using its molar extinction coefficient and normalized to protein content. The results were expressed as mM MDA per milligram of protein [69].

#### 4.5.3. PCC

Protein oxidation was determined according to the 2,4-dinitrophenylhydrazine (DNPH) derivatization method reported by Levine et al. with subsequent modifications. Aliquots of supernatant were reacted with DNPH in an acidic medium and incubated in the dark for 1 h. Proteins were precipitated with trichloroacetic acid, washed with an ethanol/ethyl acetate mixture, and dissolved in guanidine hydrochloride. Absorbance was measured at 366 nm, and carbonyl levels were calculated using the corresponding extinction coefficient and expressed as µM carbonyls per milligram of protein [70].

#### 4.5.4. SOD Activity

SOD activity was quantified following the procedure described by Misra and Fridovich. Each reaction contained a sample supernatant, carbonate buffer at alkaline pH, and adrenaline, which undergoes auto-oxidation to adrenochrome. Absorbance was monitored at 480 nm at 2 time points, and enzyme activity was determined by comparison with a standard curve. Values were normalized to protein concentration and reported as IU per milligram of protein [71].

#### 4.5.5. CAT Activity

Catalase activity was measured following the method of Radi et al. Reaction mixtures contained sample supernatant, isolation buffer, and hydrogen peroxide as the substrate. The decrease in absorbance at 240 nm over 60 s was used to calculate enzyme activity based on the extinction coefficient of H_2_O_2_. Results were expressed as µM H_2_O_2_ per milligram of protein [72].

#### 4.5.6. Total Protein

Total protein was measured following the Bradford method. Sample supernatants (25 μL) were combined with 75 μL of deionized water and 2.5 mL of Bradford reagent. After gentle mixing, the samples were incubated at room temperature for 5 min, and absorbance was measured at 595 nm. Protein content was determined using a bovine serum albumin standard curve [73].

### 4.6. Assessment of Molecular Stress Responses

For gene expression, fish were exposed under the same experimental design for 96 h. Brain, gill, gut, and liver samples were preserved in RNAlater^®^ (Qiagen, Hilden, Germany) and stored at −20 °C. Total RNA was extracted using the RNeasy^®^ Mini Kit (Qiagen, Hilden, Germany), quantified, and assessed for integrity by spectrophotometry (260/280 nm) and agarose gel electrophoresis.

Following the manufacturer’s recommended workflow, 1 µg of total RNA from each sample was used for cDNA synthesis with the QuantiTect^®^ Reverse Transcription Kit (Qiagen, Hilden, Germany). The resulting cDNA was diluted, and 2 µL of diluted cDNA were used per reaction for each target gene in the qPCR assays, following the QuantiTect^®^ SYBR Green Master Mix guidelines. Quantitative PCR was performed on a Rotor-Gene Q system (Qiagen, Hilden, Germany).

The relative expression of genes involved in antioxidant defense (*sod, cat*), redox regulation (*nfe2l2a, nfe2l2b*), detoxification (*cyp1a*), and apoptosis (*baxa, casp3, casp6, casp7, casp9*) was determined using actb2 (*β-actin*) as a reference gene. Amplification specificity was confirmed by melting-curve analysis, and relative transcript levels were calculated using the 2^−ΔΔCt^ method [74,75]. All reactions were performed in six replicates to ensure analytical reliability.

### 4.7. Statistical Data Analysis

The study assessed data distribution and variance homogeneity using the Shapiro–Wilk and Levene tests. Oxidative damage and antioxidant activity were analyzed through a two-way ANOVA. When significant effects were detected, the analysis applied Dunnett post hoc tests with Sidak correction to compare each sampling site with the control group at the corresponding exposure time. Gene expression values were normalized to the mean of the control group (control = 1). For each organ and gene, the analysis evaluated variance homogeneity with Levene’s test before performing a one-way ANOVA. When variances were homogeneous, Student’s *t*-tests with Sidak correction were used; when variances were heterogeneous, Welch’s *t*-tests with the same correction were applied. All statistical procedures were conducted in SigmaPlot (version 12.3), and statistical significance was set at *p* < 0.05.

## 5. Conclusions

Although the evaluated physicochemical parameters, except for color, were within the limits established by Mexican regulations, the analysis of heavy metals, pharmaceuticals, and biomarkers of oxidative stress in *Danio rerio* exposed to water from the Tepetitlán reservoir revealed significant pollution. Increased lipid peroxidation, protein carbonylation, and antioxidant enzyme activity were observed, along with enhanced expression of genes associated with oxidative stress and apoptosis across organs and study sites. These findings indicate severe oxidative damage in exposed organisms and highlight a risk to the health of the aquatic ecosystem. Given this scenario, preserving this water resource and ensuring its sustainable use will require coordinated actions aimed at mitigating the multiple stressors affecting it.

## Figures and Tables

**Table 1:** Concentrations of metals and pharmaceuticals at each sampling site (A–C) of the Tepetitlán reservoir.

		Sample Site			NOM-001-SEMARNAT-2021 [13]
Pollutant (μg L^−1^)		Site A	Site B	Site C	
Metal	Arsenic	16.0 ± 1.4	20.0 ± 1.8	25.0 ± 2.2	200
	Cadmium	12.0 ± 1.1	18.0 ± 1.6	160.0 ± 3.4	200
	Copper	20.0 ± 2.2	17.0 ± 1.2	21.0 ± 2.1	6000
	Chromium	20.0 ± 1.9	18.0 ± 1.5	25.0 ± 2.2	1000
	Mercury	<LOD	<LOD	<LOD	10
	Nickel	24.0 ± 2.3	<LOD	33.0 ± 2.4	4000
	Lead	<LOD	32.0 ± 2.3	17.0 ± 1.2	400
	Zinc	26.0 ± 2.4	19.0 ± 1.2	24.0 ± 2.3	20,000
Antibiotics	Dicloxacillin	469.8 ± 13.4	29.2 ± 1.9	22.3 ± 1.8	NI
	Trimethoprim	<LOD	<LOD	<LOD	NI
	Sulfamethoxazole	7.9 ± 0.8	5.6 ± 0.7	<LOD	NI
Antihyperglycemic	Metformin	57.0 ± 1.6	7.3 ± 0.2	12.6 ± 0.5	NI
Antiepileptic	Carbamazepine	36.2 ± 1.1	<LOD	<LOD	NI
Glucocorticoid	Dexamethasone	<LOD	<LOD	<LOD	NI
Non-steroidal anti-inflammatory	Diclofenac	9.8 ± 0.9	8.5 ± 0.2	<LOD	NI
	Naproxen	177.9 ± 1.4	9.2 ± 0.6	10.2 ± 0.5	NI
	Paracetamol	75.4 ± 1.2	52.2 ± 0.6	56.1 ± 1.9	NI
	Ketorolac	<LOD	<LOD	<LOD	NI
Synthetic steroid	17α-ethinylestradiol	10.1 ± 0.3	<LOD	12.8 ± 1.5	NI
Antihypertensive	Telmisartan	<LOD	<LOD	<LOD	NI

Data represent mean ± standard deviation of three independent experiments. NI = Not included; LOD = Limit of detection.

**Table 2:** Concentrations of metals and pharmaceuticals at each sampling site (D–F) of the Tepetitlán reservoir.

		Sample Site			NOM-001-SEMARNAT-2021 [13]
Pollutant (μg L^−1^)		Site D	Site E	Site F	
Metal	Arsenic	23.0 ± 1.9	11.0 ± 1.2	15.0 ± 1.2	200
	Cadmium	73.0 ± 3.5	19.0 ± 1.7	12.0 ± 1.2	200
	Copper	14.0 ± 1.2	15.0 ± 1.2	18.0 ± 1.4	6000
	Chromium	24.0 ± 2.1	28.0 ± 2.1	29.0 ± 1.8	1000
	Mercury	<LOD	<LOD	<LOD	10
	Nickel	19.0 ± 1.3	<LOD	<LOD	4000
	Lead	21.0 ± 2.0	26.0 ± 2.3	23.0 ± 2.1	400
	Zinc	15.0 ± 1.3	18.0 ± 1.4	18.0 ± 1.3	20,000
Antibiotics	Dicloxacillin	19.8 ± 1.7	31.2 ± 1.9	38.4 ± 1.5	NI
	Trimethoprim	<LOD	<LOQ	<LOD	NI
	Sulfamethoxazole	<LOD	<LOD	<LOD	NI
Antihyperglycemic	Metformin	11.7 ± 0.3	<LOQ	16.2 ± 1.6	NI
Antiepileptic	Carbamazepine	<LOD	9.7 ± 0.4	<LOD	NI
Glucocorticoid	Dexamethasone	<LOD	<LOD	<LOD	NI
Non-steroidal anti-inflammatory	Diclofenac	<LOD	7.1 ± 0.5	9.6 ± 0.4	NI
	Naproxen	<LOD	12.6 ± 0.5	<LOD	NI
	Paracetamol	52.7 ± 1.2	68.2 ± 1.9	46.1 ± 1.3	NI
	Ketorolac	<LOD	<LOD	<LOD	NI
Synthetic steroid	17α-ethinylestradiol	<LOD	13.6 ± 0.4	<LOD	NI
Antihypertensive	Telmisartan	<LOD	<LOD	<LOD	NI

Data represent mean ± standard deviation of three independent experiments. NI = Not included; LOD = Limit of detection; LOQ = Limit of quantification.

## Data Availability

The data presented in this study are available on request from the corresponding author due to institutional policies and collaborative agreements.

## Appendix A

### Appendix A.1

Table A1 summarizes the two-way ANOVA results evaluating the effects of exposure time, sampling site, and their interaction on four oxidative stress biomarkers (HPC, LPX, PCC, SOD, and CAT) measured in brain, gill, intestinal, and hepatic tissues. For each biomarker–organ combination, F-values, degrees of freedom for between-group and within-group comparisons, and the corresponding *p*-values are presented.

**Table A1:** Two-way ANOVA summary for oxidative stress biomarkers across organs, sampling sites, and exposure times.

Biomarker	Organ	Factor	F_Value	df_Between	df_Within	*p*_Value
HPC	Brain	Time	1011.447	4	70	4.18 × 10^−61^
	Brain	Site	903.645	6	70	3.17 × 10^−64^
	Brain	Time × Site	663.2872	24	70	3.54 × 10^−73^
	Gill	Time	1053.043	4	70	1.05 × 10^−61^
	Gill	Site	706.1267	6	70	1.56 × 10^−60^
	Gill	Time × Site	332.4381	24	70	9.18 × 10^−63^
	Gut	Time	436.9601	4	70	1.08 × 10^−48^
	Gut	Site	1068.085	6	70	9.8 × 10^−67^
	Gut	Time × Site	247.025	24	70	2.61 × 10^−58^
	Liver	Time	2668.853	4	70	1.09 × 10^−75^
	Liver	Site	1016.845	6	70	5.37 × 10^−66^
	Liver	Time × Site	272.8857	24	70	8.43 × 10^−60^
LPX	Brain	Time	2531.501	4	70	6.87 × 10^−75^
	Brain	Site	503.4308	6	70	1.7 × 10^−55^
	Brain	Time × Site	397.9787	24	70	1.8 × 10^−65^
	Gill	Time	773.4122	4	70	4.16 × 10^−57^
	Gill	Site	501.556	6	70	1.93 × 10^−55^
	Gill	Time × Site	519.073	24	70	1.79 × 10^−69^
	Gut	Time	379.8223	4	70	1.18 × 10^−46^
	Gut	Site	526.1881	6	70	3.76 × 10^−56^
	Gut	Time × Site	515.4378	24	70	2.28 × 10^−69^
	Liver	Time	1020.577	4	70	3.07 × 10^−61^
	Liver	Site	1554.615	6	70	2.19 × 10^−72^
	Liver	Time × Site	675.0792	24	70	1.92 × 10^−73^
PCC	Brain	Time	833.8917	4	70	3.16 × 10^−58^
	Brain	Site	1069.334	6	70	9.41 × 10^−67^
	Brain	Time × Site	324.8831	24	70	2.03 × 10^−62^
	Gill	Time	452.5032	4	70	3.33 × 10^−49^
	Gill	Site	1620.948	6	70	5.12 × 10^−73^
	Gill	Time × Site	146.163	24	70	1.72 × 10^−50^
	Gut	Time	1977.786	4	70	3.62 × 10^−71^
	Gut	Site	386.5616	6	70	1.37 × 10^−51^
	Gut	Time × Site	160.7744	24	70	6.64 × 10^−52^
	Liver	Time	632.6802	4	70	3.93 × 10^−54^
	Liver	Site	1464.618	6	70	1.73 × 10^−71^
	Liver	Time × Site	241.2268	24	70	5.93 × 10^−58^
SOD	Brain	Time	576.6267	4	70	9.21 × 10^−53^
	Brain	Site	648.6063	6	70	2.89 × 10^−59^
	Brain	Time × Site	583.114	24	70	3.13 × 10^−71^
	Gill	Time	508.1743	4	70	6.66 × 10^−51^
	Gill	Site	603.9545	6	70	3.35 × 10^−58^
	Gill	Time × Site	311.0315	24	70	9.18 × 10^−62^
	Gut	Time	11.00653	4	70	5.56 × 10^−07^
	Gut	Site	321.4433	6	70	7.01 × 10^−49^
	Gut	Time × Site	354.0208	24	70	1.04 × 10^−63^
	Liver	Time	206.7803	4	70	5.63 × 10^−38^
	Liver	Site	553.4661	6	70	6.66 × 10^−57^
	Liver	Time × Site	102.728	24	70	2.7 × 10^−45^
CAT	Brain	Time	457.5052	4	70	2.3 × 10^−49^
	Brain	Site	456.8902	6	70	4.66 × 10^−54^
	Brain	Time × Site	195.4359	24	70	8.26 × 10^−55^
	Gill	Time	390.9801	4	70	4.5 × 10^−47^
	Gill	Site	630.0716	6	70	7.83 × 10^−59^
	Gill	Time × Site	278.6461	24	70	4.1 × 10^−60^
	Gut	Time	191.0687	4	70	7.11 × 10^−37^
	Gut	Site	1077.107	6	70	7.32 × 10^−67^
	Gut	Time × Site	275.7849	24	70	5.85 × 10^−60^
	Liver	Time	559.3934	4	70	2.58 × 10^−52^
	Liver	Site	1182.338	6	70	2.9 × 10^−68^
	Liver	Time × Site	290.9659	24	70	9.2 × 10^−61^

### Appendix A.2

The table presents the results of Levene’s test and one-way ANOVA for each gene and organ, including the Levene *p*-value, F statistic, degrees of freedom, and ANOVA *p*-value. These values summarize whether gene expression differed among sampling sites and support the statistical comparisons shown in the main figures.

**Table A2:** Summary of Levene’s test and one-way ANOVA results for each gene and organ.

Organ	Gene	Levene_p	F	df_Between	df_Within	p_ANOVA
Brain	*sod*	0.400495	1.918523	6	35	0.105223
Brain	*cat*	0.312834	1.501969	6	35	0.206297
Brain	*bax*	0.379454	2.855224	6	35	0.022717
Brain	*cyp1a1*	0.749604	2.27221	6	35	0.058883
Brain	*nrf2a*	0.963232	3.370097	6	35	0.009966
Brain	*nrf2b*	0.481278	4.265592	6	35	0.002512
Brain	*casp3*	0.007707	4.035853	6	35	0.003552
Brain	*casp7*	0.347768	3.628902	6	35	0.006641
Brain	*casp6*	0.583416	4.209386	6	35	0.002733
Brain	*casp9*	0.980062	2.851421	6	35	0.022857
Gill	*sod*	0.061263	0.943663	6	35	0.476776
Gill	*cat*	0.146639	0.714545	6	35	0.640318
Gill	*bax*	0.11687	0.844367	6	35	0.544592
Gill	*cyp1a1*	0.613362	9.153152	6	35	4.86 × 10^−6^
Gill	*nrf2a*	0.001489	2.217584	6	35	0.064412
Gill	*nrf2b*	0.293253	8.73447	6	35	7.71 × 10^−6^
Gill	*casp3*	0.171732	10.40048	6	35	1.31 × 10^−6^
Gill	*casp7*	0.05149	10.58826	6	35	1.08 × 10^−6^
Gill	*casp6*	0.062733	3.398564	6	35	0.009528
Gill	*casp9*	0.112206	7.98309	6	35	1.82 × 10^−5^
Gut	*sod*	0.088235	0.967589	6	35	0.46128
Gut	*cat*	0.033815	1.420702	6	35	0.234529
Gut	*bax*	0.448129	0.846295	6	35	0.543226
Gut	*cyp1a1*	0.081412	1.27535	6	35	0.293792
Gut	*nrf2a*	0.074585	1.598315	6	35	0.176901
Gut	*nrf2b*	0.433786	3.14797	6	35	0.014184
Gut	*casp3*	0.002089	12.9058	6	35	1.21 × 10^−7^
Gut	*casp7*	0.003174	3.399741	6	35	0.00951
Gut	*casp6*	0.604781	2.2408	6	35	0.062001
Gut	*casp9*	0.814872	11.07555	6	35	6.68 × 10^−7^
Liver	*sod*	0.429915	0.477841	6	35	0.820147
Liver	*cat*	0.050755	1.188851	6	35	0.33489
Liver	*bax*	0.132257	2.019636	6	35	0.089159
Liver	*cyp1a1*	0.711331	1.566393	6	35	0.186178
Liver	*nrf2a*	0.873615	4.161782	6	35	0.002936
Liver	*nrf2b*	0.32901	3.499569	6	35	0.008129
Liver	*casp3*	0.468345	3.88097	6	35	0.004499
Liver	*casp7*	0.119319	3.851747	6	35	0.004705
Liver	*casp6*	0.605988	4.190121	6	35	0.002814
Liver	*casp9*	0.582768	2.582918	6	35	0.03538
